# Long-Term Consequences of Placental Vascular Pathology on the Maternal and Offspring Cardiovascular Systems

**DOI:** 10.3390/biom11111625

**Published:** 2021-11-03

**Authors:** Marisa Benagiano, Salvatore Mancuso, Jan J. Brosens, Giuseppe Benagiano

**Affiliations:** 1 Department of Experimental and Clinical Medicine, University of Florence, 50134 Florence, Italy; 2 Department of Life Sciences, Catholic University of Rome, 00168 Rome, Italy; salmancuso71@gmail.com; 3 Division of Biomedical Sciences, Warwick Medical School, Coventry CV4 7HL, UK; J.J.Brosens@warwick.ac.uk; 4 Department of Maternal and Child Health, Gynecology and Urology, Sapienza University of Rome, 00185 Rome, Italy; pinoingeneva@bluewind.ch

**Keywords:** cardiovascular diseases, great obstetrical syndromes, hypertensive disorders of pregnancy, preeclampsia, trans-generational effects

## Abstract

Over the last thirty years, evidence has been accumulating that Hypertensive Disorders of Pregnancy (HDP) and, specifically, Preeclampsia (PE) produce not only long-term effects on the pregnant woman, but have also lasting consequences for the fetus. At the core of these consequences is the phenomenon known as defective deep placentation, being present in virtually every major obstetrical syndrome. The profound placental vascular lesions characteristic of this pathology can induce long-term adverse consequences for the pregnant woman’s entire arterial system. In addition, placental growth restriction and function can, in turn, cause a decreased blood supply to the fetus, with long-lasting effects. Women with a history of HDP have an increased risk of Cardiovascular Diseases (CVD) compared with women with normal pregnancies. Specifically, these subjects are at a future higher risk of: Hypertension; Coronary artery disease; Heart failure; Peripheral vascular disease; Cerebrovascular accidents (Stroke); CVD-related mortality. Vascular pathology in pregnancy and CVD may share a common etiology and may have common risk factors, which are unmasked by the “stress” of pregnancy. It is also possible that the future occurrence of a CVD may be the consequence of endothelial dysfunction generated by pregnancy-induced hypertension that persists after delivery. Although biochemical and biophysical markers of PE abound, information on markers for a comparative evaluation in the various groups is still lacking. Long-term consequences for the fetus are an integral part of the theory of a fetal origin of a number of adult diseases, known as the Barker hypothesis. Indeed, intrauterine malnutrition and fetal growth restriction represent significant risk factors for the development of chronic hypertension, diabetes, stroke and death from coronary artery disease in adults. Other factors will also influence the development later in life of hypertension, coronary and myocardial disease; they include parental genetic disposition, epigenetic modifications, endothelial dysfunction, concurrent intrauterine exposures, and the lifestyle of the affected individual.

## 1. Introduction

During the first half of a normal pregnancy, a unique phenomenon takes place on the maternal side of the forming placenta: the physiological spiral artery remodeling. It is important to stress that this fundamental process starts during the secretory phase of a menstrual cycle, when the endometrium transforms into a well-vascularized tissue and vascular permeability increases. In addition, stromal cells transform themselves into decidual cells, there is a leukocyte invasion and angiogenesis [1]. In the event of pregnancy, this process progresses with additional angiogenesis and the beginning of vascular adaptation [2].

The most characteristic aspect of spiral artery remodeling is cytotrophoblast invasion of spiral arteries [3]. However, it has been documented that remodeling starts before interactions between vascular wall components and cytotrophoblast begin [4]. The process is primed by the surrounding macrophages and tissue-resident CD56 super-bright cells, termed ‘decidual’ natural killer cells, phenotypically and functionally distinct from conventional uterine and circulating natural killer cells [5,6]. Intravascular trophoblast first ‘plugs’ the terminal spiral arterioles, thereby enabling the conceptus to develop during the critical period of organogenesis under low oxygen tension. During this phase, profuse glandular secretions, rich in growth factors, lipids and carbohydrates, nourish the placenta and fetus. Towards the end of the first trimester, the trophoblast plugs-in the spiral arteries and progressively dislocates, allowing gradual perfusion of the intervillous space [7]. There follows progressive extravillous trophoblast invasion of decidual and inner myometrial spiral arteries and their transformation by dilatation, loss of the elastica, and disorganization of smooth muscle cells into low-resistance vessels [8].

Brosens et al. [9] summarized and classified pathological conditions that may alter this process that goes under the name defective, deep placentation [10]. These are: Preeclampsia (PE), early-onset Fetal Growth Restriction (FGR), Chronic hypertension with superimposed PE, Chronic hypertension with FGR, Abruptio Placentae, Partial Preterm Labor with intact membranes, Preterm Premature Rupture of Membranes (PPROM), FGR without hypertension [8,9].

In the substantial or partial absence of spiral artery remodeling, uterine vessels in the inner portion of the myometrium (coined the Junctional Zone) are prone to develop a specific pathology, acute atherosis [10]. Its characteristics have been recently described [11], revisiting a classic thesis on the subject [12]; the lesion is defined by the presence of fibrinoid necrosis, subendothelial macrophage foam cells (FC), and perivascular lymphocytic infiltration. Significant amounts of intracellular and extracellular lipids can accumulate in the decidua basalis, often extending into the superficial layer of the myometrium. This phenomenon, termed diffuse lipid infiltration, has been found also in post-term pregnancies but, unfortunately, has been neglected in the context of placental bed pathophysiology.

The first description of what we call today acute atherosis was probably that made by Hertig in 1945 in women with PE; at the time, it was called hypertensive albuminuric toxemia [13]; in 1950, his group further described vascular placental features in pathological pregnancies [14].

The same year, Zeek and Assali [15] detailed the presence of this pathology in preeclamptic toxemia, specifying that in hypertensive pregnancies (a situation they called eclamptogenic toxemia of pregnancy) these lesions are associated to hyperplastic arteriolar sclerosis of spiral arteries. Acute atherosis consists of an accumulation of macrophages filled with lipids (i.e., FC), thickening of the tunica intima and fibrinoid necrosis of the tunica media [16,17]. An acute vessel necrosis associated with intramural infiltrates was described by Lendrum et al. in 1978 as a form of plasmatic vasculosis [18].

Acute atherosis represents a conspicuous vasculopathy in pregnancy [19] and its incidence and pathological significance continues to be debated [20,21,22].

The term atherosis refers to vascular disease at the feto-maternal interface and is specific of PE. When it extends to thetract in the myometrial Junctional Zone, it becomes indistinguishable from atherosclerosis, the primary driver of Cardiovascular Diseases (CVD). CVDs have been defined by WHO [23] and include: hypertension; coronary heart disease; cerebrovascular disease; peripheral vascular disease; heart failure; rheumatic heart disease; congenital heart disease; cardiomyopathies.

Contrary to popular belief, the atherosclerotic process begins in the fetus where, its earliest lesion, the fatty streak has been demonstrated. The process is greatly enhanced by the presence of hypercholesterolemia during pregnancy due to the presence of oxidized Low Density Lipoproteins (oxLDL) [24]. The inflammatory nature of atherosclerosis has been known for centuries [25,26] and it has been demonstrated to be a T helper type 1 lymphocytes mediated disease [27,28]. Ample proof of this reality was presented more than 20 years ago with the final results of the so-called Bogalusa Heart Study [29] of autopsies on 204 young persons (2 to 39 years), who had died from various causes. This investigation found that the extent of fatty streaks and fibrous plaques in the aorta and coronary arteries increased with age, with a much stronger association in the coronary arteries (r = 0.60, *p* < 0.001) than in the aorta (r = 0.23, *p* = 0.03). A report published in 1992 [30] detailed sex and ancestry in 150 young people, aged 6 to 30 years: aorta intimal surface involvement with fatty streaks varied from 0 to 71%, and was more prevalent in blacks than in white people (*p* > 0.001).

A number of factors can determine subsequent progression: body mass index, central obesity, waist circumference non-high-density lipoprotein cholesterol, blood pressure, blood glucose, and smoking status [31,32]. Good examples of early onset atherosclerosis are type 1 diabetes and accelerated atherosclerosis occurring in blood vessels of transplanted organs. [33,34]. The condition has a very strong genetic common pathway, justifying joining together all major pregnancy complication, as evidenced by the heritable nature of the disease [35]. Neuropeptide Y (NPY) is a biomolecule with various important functions. It is the most abundant peptide in the heart and brain, and is produced by sympathetic neurons, endothelial cells (ED), and platelets. Moreover, it plays a role in sympathetic nerve stimulation through co-release with norepinephrine, immune function, regulation of food consumption, modulation of heart rate, vasoconstriction, coronary blood flow and ventricular function. An increased risk of coronary artery disease can be conferred by neuropeptide Y gene polymorphisms [36].

An important feature of placental bed atherosclerosis is that it occurs to a greater extent and at a lower level of hypertension in the placental bed than in other organs [37].

In this chapter, we summarize and try to evaluate explore the available evidence that pregnancy disorders caused by defective spiral artery remodeling are sentinel risk markers for future CVD in the mother as well as her baby. It must be stressed that, whereas there is an abundance of published reports on the subject, it is still extremely difficult to draw a complete picture of the situation.

## 2. Cardiovascular and Metabolic Consequences of the Great Obstetrical Syndromes

The expression “Great Obstetrical Syndromes” was coined to call attention to the idea that etiological heterogeneity was followed by a common pathway for PE, intrauterine growth restriction, preterm labor, PPROM, late spontaneous abortion, and Abruptio Placentae [38]. In addition, Brosens et al. [39] suggested that cyclic decidualization, followed by menstruation serves as a preconditioning mechanism to prepare the uterus for deep placentation and that cyclic menstruation may have a critical role in protecting uterine tissues from the inflammatory and oxidative stress associated with it.

Today, there is ample evidence that defective deep placentation is at the heart of the spectrum of pregnancy complications, being present in virtually every major obstetrical syndrome [40]. On the one hand, profound placental vascular lesions can later induce adverse consequences for the pregnant woman’s entire arterial system. On the other, restriction of placental growth and function can, in turn, cause a restricted blood supply to the fetus, with long-lasting consequences.

Interestingly, some early studies reported no adverse consequences of hypertensive disorder in pregnancy (HDP), PE, or eclampsia on long-term maternal health [41,42,43]. Others, however, found a correlation, especially in the event of a young primigravida [44].

It seems that the first report of a correlation between increased blood pressure (BP) during pregnancy and subsequent higher BP in mothers and their offspring was that by Langford and Watson in 1980 [45]. They were also the first to detect a difference between male and female offspring, with the increase being observed in daughters.

A more recent review into PE, with focus on impact on the fetus, short and long-term outcome of offspring, and long-term outcome of women with a history of PE has been published in 2016 [46].

## 3. Consequences for the Pregnant Woman

In spite of a fairly large number of investigations on the short- and long-term consequences of HDP, a 2017 systematic review and meta-regression analysis by Groenhof et al. of studies aimed at CVD prevention following HDP concluded that, unfortunately, there is no way to “point out a time point to commence screening for cardiovascular risk factors in women after an HDP” l [47]. In fact, the only clearly established fact pointed out by existing studies is a link between PE and an increased risk of CVD and, as stated by Muijsers [48], “for high-risk women, PE may be considered a first cardiovascular event that requires secondary prevention and appropriate follow-up”.

In 2002, an editorial in the BMJ [49] summarized the evidence available at the time, linking the Great Obstetrical Syndromes, and in particular HDP, to adverse long-term health consequences for both the mother and her child (Table 1).

A large number of publications have reported the results of prospective cohort investigations on a possible association between HDP and risk of CVD later in life. Here, we limit our description to results obtained in one prospective cohort study and in four systematic reviews and metanalyses.

In 2019, Haug et al. [50] published the results of a prospective cohort trial of 23,885 parous women from a Norwegian County. They found that women aged between 40 and 70 years, with history of HDP, had an increased risk of CVD compared with women with normal pregnancies (Hazard ratio (HR): 1.57; 95% confidence interval (95%CI): 1.32–1.87), but not at older age (β = 0.98; 95%CI: 0.96–1.00; *p* for interaction by age = 0.01). In addition, BP and body mass index were associated with up to 77% of the excess CVD risk in women with history of HPD. In conclusion, an increased risk of CVD seemed associated with history of hypertension during a gestation, as well as with conventional cardiovascular risk factors, indicating that these represent specific targets for CVD prevention.

As mentioned, a number of systematic reviews and metanalyses have investigated the consequences of PE and pregnancy hypertension for the future cardiovascular health. In particular, three systematic reviews examined whether PE carries an increased risk of CVD later in life.

In 2007, Bellamy et al. [51] found an increased future risk for:

(a) Hypertension: Relative risk (RR) = 3.7, 95% CI: 2.70–5.05, after 14.1 years weighted mean follow-up.
(b) IHD: RR = 0.16, 95% CI: 1.86–2.52, after 11.7 years.
(c) Stroke: RR = 1.81, 95% CI: 1.45–2.27, after 10.4 years.
(d) Venous thrombo-embolism: RR = 1.79, 95% CI: 1.37–2.33, after 4.7 years.

In 2013, Brown et al. [52] found an increased future risk for:

(a) Fatal or diagnosed CVD: Odds Ratio (OR) = 2.28, 95% CI: 1.87–2.78.
(b) Cerebrovascular accident: OR = 1.76, 95% CI: 1.43–2.21;
(c) Hypertension: RR = 3.13, 95% CI: 2.51–3.89.

They also found that pre-term delivery in women with preeclampsia was not associated with an increased risk of future CVD: RR = 1.32, 95% CI: 0.79–2.22.

In 2020, Wu et al. [53] evaluated 66 cohort and 7 case-control studies, involving >13 million women and found an increased future risk for:

(a) Any CVD: RR = 1.80, 95%CI: 1.67–1.94.
(b) Coronary artery disease: RR = 1.66, 95% CI: 1.49–1.84
(c) Heart failure: RR = 2.87, 95% CI: 2.14–3.85.
(d) Peripheral vascular disease: RR = 1.60, 95% CI: 1.29–2.00.
(e) Stroke: RR = 1.72, 95% CI: 1.50–1.97.
(f) CVD-related mortality: RR = 1.78, 95% CI 1.58–2.00.
(g) Hypertension: RR = 3.16, 95% CI: 2.74–3.64)

In conclusion, all three metanalyses found an association between a pregnancy complicated by PE and an increased risk of a spectrum of vascular disease in later life. The increase varied between two and five fold.

A fourth metanalysis concentrated on a possible increased risk of abnormal biochemical cardiovascular risk factors following an HDP vs. a normal gestation [54]. Results are shown in Table 2.

Finally, Brouwers et al. [55], examined the risk of CVD in women with recurrent PE, compared to women who had one or more normal pregnancies following a preeclamptic pregnancy. They observed that recurrent PE was consistently associated with an increased pooled risk ratio of hypertension (RR: 2.3; 95% CI: 1.9–2.9), IHD (RR: 2.4; 95% CI: 2.2–2.7), heart failure (RR: 2.9; 95% CI: 2.3–3.7), cerebrovascular accident (RR: 1.7; 95% CI: 1.2–2.6) and hospitalization due to CVD (RR: 1.6; 95% CI 1.3–1.9). They also found investigations indicating a beneficial effect on venous thromboembolism, atherosclerosis and mortality for CVD, but the data could not be pooled.

An interesting investigation by Smith et al. [56] examined whether pregnancy resulting in low body weight infants increases the risk of subsequent ischemic heart disease (IHD) in the mother. They found that maternal risk of admission or death for IHD was associated with delivering a baby in the lowest birthweight quintile for gestational age (adjusted hazard ratio (aHR): 1.9; 95% CI: 1.5–2.4), preterm birth (PTB) (aHR: 1.8; 95%CI: 1.3–2.5), and PE (aHR: 2.0; 95% CI: 1.5–2.5). The associations were additive; women with all three characteristics had a seven times greater risk of IHD admission or death (95%CI: 3.3–14.5) than controls.

### 3.1. Pathogenetic Mechanisms of Delayed Effects of Preeclampsia

Brosens et al. [57] recently summarized the pathogenetic mechanisms leading to atherosclerosis of spiral arteries in the placental bed, concluding that these lesions are caused by the same cellular and molecular mechanisms active in other vascular systems of the body, namely, an inflammatory condition of the arterial wall. In this regard, it has been suggested that women who develop PE and those developing CVD may have common risk factors, which are unmasked by the “stress” of pregnancy [58]. This is supported by an investigation comparing subjects with decidual atherosclerotic vasculopathy with cases without this condition, 7 months postpartum. The study showed that women with a preceding PE and atherosclerotic lesions of uterine vessels had increased cardiovascular risk [59].

Indeed, immunopathological involutions have been demonstrated across the arterial tree, including carotid arteries, iliac, and popliteal arteries [27] and it seems plausible that these complex abnormalities may occur also in the myometrial spiral arteries during postpartum [60,61].

The topic has been further explored by Ying et al. [62] who described various pathophysiological hypotheses that may link PE and pregnancy hypertension to maternal CVD later in life. A first possibility is that there are common predisposing risk factors and therefore that both conditions represent manifestations of the same pathophysiological processes at different times in a woman’s life [63,64,65]. Alternatively, a second option is that the future occurrence of a CVD may be the consequence of endothelial dysfunction (ED) generated by pregnancy-induced hypertension that persists after delivery [66,67,68,69,70]. This hypothesis is controversial and not corroborated by other studies [71].

Osol & Bernstein [72] detailed evidence for the two opposite theories, namely, that alterations occurring in pregnancy may be at the origin of the increased CVD risk, and that, on the contrary, PE might be the manifestation of a maternal phenotype with some predisposition to CVD. A recent review [73] described the range of increased risks associated with pregnancy complications: hypertension, left ventricular hypertrophy/dysfunction, vascular and renal dysfunction. The authors stressed that the vascular abnormalities that are part of the picture in pathological pregnancies, such as cardiac microvascular dysfunction and heart failure with preserved ejection fraction, will impact on a woman’s life as she ages. They concluded that vascular pathology in pregnancy and CVD share a common etiology. One such mechanism may involve altered epigenetic programming of specific tissues, induced by excessive oxidative stress (OS) [9,74,75]. Whether these changes can influence the risk of CVD later in life is not yet known.

Finally, Turbeville & Sasser [76] have summarized the disease-specific molecular mechanisms that may help explain the long-term risks following PE: among them, postpartum perturbations of physiological pathways, including sodium and angiotensin II sensitivity, sympathetic activation, and ED. Specifically, they report studies showing that, following HDP, women have a significantly higher index of salt sensitivity, with a significantly increased pressor response to high-salt diet. This phenomenon seems related to an increase in vasopressin production and circulation. With regard to angiotensin II, PE is also accompanied by a significant decrease in its levels, an increased vascular response and a reduction in endothelium- and nitrous oxide (NO)-dependent vasodilatation. Last but not least, ED persisting up to 5–8 years has been demonstrated following a preeclamptic pregnancy.

### 3.2. Biomolecules Involved in the Pathogenesis of Long-Term Effects

A fairly large number of biomarkers of the consequences of HDP for CVD have been identified. Unfortunately, investigations into biomolecules that could be markers for the long-term consequences of HDP are lacking. For this reason, there is a strong need forfuture perspective studies in large cohorts of women with placental vascular bed pathology to search for new cardiovascular biomarkers that could be associated with postpartum CVD risk of mothers and offspring and to develop prognostic models for adequately stratifying the risk of developing CVD in later life.

A series of biomolecules are involved in the pathogenesis of the placental bed pathology and they are strictly related to those involved in the pathogenesis of CVD.

Herein, we present and describe various pathways for which there are active components involved in both processes. Although a series of biomolecular parameters have been identified and measured, to our knowledge, at present, there are no specific and well-established biomarkers with a strong predictive value of future CVD outcome. Nevertheless, a series of investigations point to several parameters with putative predictive value which need to be more extensively investigated.

#### 3.2.1. Inflammation and the Role of Inflammatory Biomolecules

As extensively demonstrated in the literature since the early 90s, atherosclerosis is now defined as an inflammatory disease [77]. A great number of biomolecules such as cytokines, growth factors, vasoregulatory molecules and lipids along with small molecules like nitric oxide participate in this process and they eventually play different roles at different stages of the disease, regardless of the primary cause that started the process [26].

A similar scenario, still under intensive study by the scientific community, can be found for placental bed pathology, stressing the importance of the role of immune system, which not only contributes to its pathogenesis, but is also considered the primary link to future CVD risk. It has been suggested that PE is a three-stage disorder with the primary pathology being an excessive or atypical maternal immune response. This would impair the placentation process leading to chronic oxidative stress in the placenta and finally, to diffuse maternal ED [78]. Indeed, cytokines released from the ischemic placenta trigger a systemic oxidative and inflammatory state [79]. Selected evidence highlights postpartum inflammatory changes regarding circulating markers of inflammation in women who experienced placental vascular bed pathology syndromes, underling the importance of the proinflammatory phenotype as a common feature that could link pregnancy vascular bed disorders to future CVD in these subjects [70,79].

#### 3.2.2. In Vivo Acute-Phase Response Markers

Among acute phase response biomolecules, C-reactive protein (CRP) is one of the most characterized in literature and substantial evidence glimpses its possible role as a biomarker able to link placental vascular bed pathology to CVD. CRP is higher in women with prior PE vs. controls, as demonstrated in a systemic acute-phase response study after early-onset PE [80]. Hauspurg et al. demonstrated significant high sensitivity CRP (hs-CRP) abnormalities, in the first-year post-partum among women with HDP and normotensive ones [81]. Furthermore, CRP is an important inflammation biomarker in metabolic syndrome where visceral fat produces a chronic state of inflammation leading to CVD [82,83]. The Canakinumab Anti-Inflammatory Thrombosis Outcomes Study (CANTOS) specifically targeted interleukin-1β, a proinflammatory cytokine that plays multiple roles in the development of athero-thrombotic events, in patients with a history of myocardial infarction and a hs-CRP level of 2 mg or more per liter had significantly reduced hs-CRP levels from baseline, as compared with placebo, without reducing the LDL cholesterol level. The cytokine-based therapy resulted in a significantly lower incidence of recurrent cardiovascular events than placebo, suggesting that reducing inflammation without affecting lipid levels may reduce the risk of CVD [84].

#### 3.2.3. Metabolic Syndrome Biomolecules

**Figure 1:**
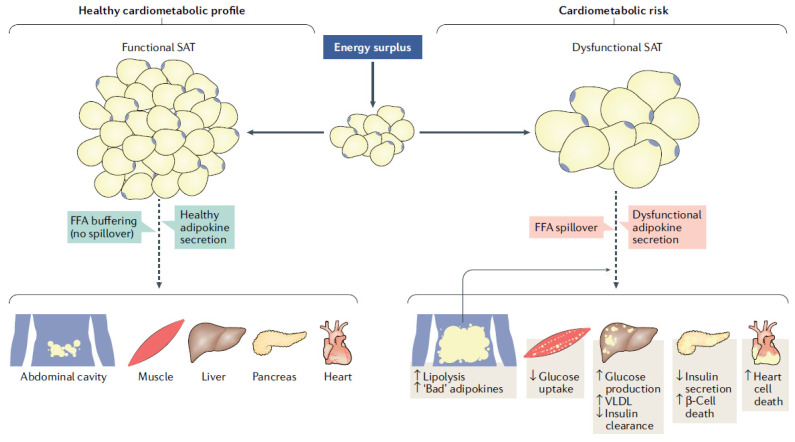
Cardiometabolic risk from dysfunctional adipose tissue is associated with altered adipokines and proinflammatory cytokines production which leads to a series of metabolic dysfunctions. Reproduced from: Ross et al., 2020.

Metabolic syndrome is characterized by an overwhelming increase in the prevalence of obesity and associated metabolic disturbances such as insulin-resistance, type 2 diabetes, hepatic steatosis, HBP, cognitive impairment and CVD. Visceral fat [85], also defined as ‘inflammatory fat tissue’, is the main driver of this process. It is able to produce a chronic pro-inflammatory state responsible for profound metabolic changes and homeostasis perturbation, which lead to a series of pathological scenarios, through pro-inflammatory cytokines and altered adipokines, including an accelerated aging process. [32,86,87]. Adipose tissue can now be considered as an endocrine organ orchestrating crucial interactions with vital organs and tissues such as brain, liver, skeletal muscle, heart and blood vessels themselves (Figure 1). Thus, the evidence suggests that adipose tissue quality/function is as important, if not more so, as its amount in determining the overall health and CV risks of overweight/obesity [88]. As previously pointed out overweight and obese women who develop hypertension during pregnancy are at higher risk of developing hypertension and biomarkers abnormalities in the first-year post-partum [81].

#### 3.2.4. Oxidative-Stress Biomolecules

Oxidative stress is one of the major causes of biological structures’ damage. When highly reactive O_2_ free radicals (OFRs) and other reactive O_2_ species (ROS) production exceeds the natural cellular protection, indiscriminate damage can occur to proteins, lipids and DNA [89]. In this connection, there is evidence that oxidative-stress, or an imbalance in the oxidant/antioxidant activity in utero–placental tissues, plays a pivotal role in the development of placental-related diseases. Jauniaux et al. described how these molecules can profoundly affect pregnancy outcome throughout the whole gestation and, in particular in PE, leading to diminished perfusion of the intervillous space which results in hypoxia and in a low-grade ischaemia–reperfusion type of injury in the placenta. OFRs lead to the formation of lipid peroxides which alter cell membranes by increasing incorporation of cholesterol and oxidized free fatty acids (FFAs) and low-density lipoproteins [78]. As for atherogenesis [90], chronic oxidative stress in the placenta leads to impaired circulation and diffuse maternal endothelial cell dysfunction. Abnormal endothelial function exemplified by increased circulating levels of fibronectin and von Willebrand factor, markers of endothelial cell injury, is found in women with PE. Most studies point to a damaged endothelium deficient in its hemostatic function, ultimately producing vasoconstriction. Decreased production of NO, prostacyclin and increased production of thromboxane, endothelin and increased vascular reactivity to Ang II in preeclamptic women also suggest abnormal endothelial function [91].

Furthermore, the placental bed of women with PE is infiltrated with activated macrophages which release biomolecules that are capable of reducing trophoblastic invasiveness and even initiating apoptosis [92].

Activated decidual leukocytes may also be a major source of OFRs, and they may produce cytokines which increase the inflammatory reaction [93]. Signs of severe oxidative stress are evident in term placentae of infants born to mothers with PE. Yang et al. demonstrated that umbilical cord fibroblasts derived from PE infants are intrinsically less able to respond to acute oxidative stress than controls, and this phenotype is retained over many cell doublings. Whether the basis of this vulnerability is genetic or epigenetic and how it pertains to trophoblast development remains unclear, but this finding may provide a clue to the basis of the early onset, usually severe, form of PE [94].

#### 3.2.5. Proangiogenic and Antiangiogenic Biomolecules

Preeclamptic placenta is characterized by the over-expression of anti-angiogenic factors that inhibit the normal function of pregnancy-related proangiogenic factors, including vascular endothelial growth factor (VEGF) and placental growth factor (PlGF) [95]. Fundamental new information has been obtained in a Scandinavian genome-wide association study by McGinnis et al. in 2017 [96]. This investigation identified the first genome-wide significant susceptibility locus (rs4769613; *p* = 5.4 × 10^−11^) near the FLT1 gene encoding Fms-like tyrosine kinase 1 on chromosome 13. This finding is of importance because soluble Fms-like tyrosine kinase 1 (sFlt-1), the placental isoform of this enzyme, is implicated in the pathogenesis of PE. Commenting on this discovery, Gray et al. [97] mentioned that the relevance of the finding lies on the fact that sFlt-1, a spliced variant of the VEGF receptor, exerts antiangiogenic activity by inhibiting signaling of pro-angiogenic factors. In 2004, Levine et al. pointed out that increased levels of sFlt-1, which binds PlGF and vascular VEGF, and reduced levels of PlGF predict the subsequent development of PE [98]. sFlt-1 has also been investigated as an angiogenic marker along with PlGF and soluble Endoglin (sEN), in order to provide new insights on a woman’s response to the cardiovascular challenges of pregnancy. This, because rising circulating levels of sEN and ratios of sFlt1:PlGF signal the onset of PE.

Additional data also suggest that sEN and sFlt1, both causing ED by different mechanisms, may contribute to PE syndrome [99]. Pathophysiological implications of lower PlGF concentrations in mid-pregnancy described by Benschop et al. in 2019 might provide insight into the identification of pathways contributing to greater cardiovascular risk factor burden [100]. As Turbeville et al. pointed out, these newer angiogenesis related biomarkers such as PlGF and PlGF-to-sFlt-1 ratio are gaining both predictive and diagnostic utility and may possibly also be helpful in distinguishing preeclampsia from other causes of hypertension in pregnancy [76]. However, Neuman et al. indicated that the proangiogenic PlGF, the antiangiogenic sFlt-1 and sFlt-1/PlGF ratio are not associated with hypertension one-year post-partum. Their data indicate that these markers are not suitable for the prediction of hypertension and the guidance of the follow-up of women with previous PE [101].

#### 3.2.6. Altered Placental Biomolecules

There is a keen interest in using placental biomolecules as possible biomarkers at birth to predict postnatal health outcomes.

The problem is that, as shown by Wilson et al. [102], while specific molecular changes in the placenta may be associated with specific pathologies, it may not be possible to identify them through the presence of relevant biomarkers in the maternal blood.

Altered placental morphology has been linked to various disorders, like high blood pressure in children and adults, and chronic heart failure in adults. The possible goal of protein-based marker, DNA and RNA marker in the maternal blood is to link placental phenotype to clinical outcomes [103].

### 3.3. Biophysical Markers Involved in the Pathogenesis of Long-Term Effects

In 2019, Kirollos et al. [104] summarized available evidence with respect to maternal vascular structure and function in association with a pre-eclamptic pregnancy.

They reported on the following biophysical markers:

(a) Carotid Intima-Media Thickness (CIMT): Conflicting evidence raises the possibility that vascular structural changes may manifest as a result of hypertension, possibly as an adaptive response to increased arterial stress. Discrepant findings have been reported in the post-partum with respect to the persistence of increased CIMT. The evidence of long-term changes comes from a meta-analysis of women with a history of PE up to 10 years postpartum, showing greater CIMT in the PE group: 0.18 mm (95% CI, 0.05–0.30 mm) [105].
(b) Cardiac Computed Tomography and Calcium Score: There is evidence in the post-partum period of a strong association between PE and vascular structural changes. HDP is significantly associated with coronary artery calcification even after adjusting fo serum creatinine levels, urinary albumin/creatinine ratio, menopause and diabetes status and antihypertensive medication use.
(c) Retinal Microvasculature: An investigation found that during PE there is a significant decrease in central retinal artery and vein equivalent diameters: 1 year postpartum, the decrease persisted [106].
(d) Flow Mediated Dilatation (FMD): Evaluation through FMD, indicates that the ED seen in PE is likely to persist after delivery, at least over the short period (up to 6 months).
(e) Pulse Wave Velocity (PWV): A consistent finding is that preceding onset of PE, there is an increase in PWV, lasting at least up to 2–3 years postpartum.

In 2011, the American Heart Association formally recognized history of PE as an independent risk factor for CVD and followed with similar recommendations concerning stroke in 2014. These mechanisms, particularly the activation of immune cells, may result in a lasting physiological memory that contributes to the long-lasting consequences in women with a history of PE [76].

## 4. Consequences for the Offspring

The theory of a fetal origin of a number of adult diseases was first proposed by Barker in 1990 [107], expanding earlier findings by Forsdahl [108], that adverse living conditions during childhood increase the risk of ischemic heart disease later in life. Barker proposed that research should be redirected towards the intrauterine environment, rather than the post-natal period, believing that a number of adult pathological conditions could be ascribed to events occurring during gestation. As pointed out by Romero [109], intrauterine malnutrition represents a significant risk factor for the development of chronic hypertension, diabetes, stroke and death from coronary artery disease in adults. Possible mechanisms for these effects are permanent changes in lipid metabolism and coagulation cascade.

During the 1990s, Barker and his group presented evidence for a variety of intergenerational effects of an abnormal pregnancy, such as in utero growth and blood pressure in childhood and adult life [110]; a correlation between fetal and placental size and risk of hypertension in adult life [111]; between fetal length, ponderal index, head circumference and the risk hypertension in adult life [112]; and between fetal nutrition and CVD in adult life [113]. They also showed that babies with low body weight had higher systolic blood pressure, starting during adolescence, and that this relationship became more pronounced with increasing age; thus, the impact of maternal hypertension during fetal life becomes amplified in her offspring from infancy to old age [114].

Consequences of FGR are not restricted to the cardiovascular system. Back in 1988, Brenner et al. [115] proposed that adverse intrauterine conditions may reduce the number of nephrons in the fetal kidney, contributing to later hypertension. This phenomenon may have as a consequence a decreased sodium excretion due to a reduced filtration surface area. The risk of chronic kidney disease would then be increased because renal adaptive capacity is reduced. Recently, this concept has been applied to a “Developmental Approach to the Prevention of Hypertension and Kidney Disease” [116].

In 2016, Pinheiro et al. [117] found that HDP had an overall negative impact on offspring’s cardiovascular, immune and neurological health. The most reliable associations they found were: HDP and higher offspring’s BP; PE and offspring’s lower cognitive functioning.

### 4.1. Hypertension

Following the pioneering work of Barker’s Group, research was initiated on the possible disturbance of BP homeostasis in the offspring of women in whom pregnancy was complicated by HDP and PE. Already thirty years ago, Palti and Rothschield [118] studied, in offspring of women with HDP, the presence of higher BP values at age 6. They found a mean systolic BP (SBP) in affected offspring of 101.3 ± 10.2 mmHg compared to 99.8 ± 9.5 mmHg in controls. The mean diastolic BP (DBP) was significantly higher among the cases than among the controls (66.2 ± 8.3 mmHg vs. 63.9 ± 8.0 mmHg, *p* = 0.03). Another early investigation, carried out in offspring aged 17, found that SBP was greater than 140 mmHg in 6.9% of the girls and 11.0% of boys in the affected group, compared with 2.9 and 9.9%, respectively, in the controls. Using multiple logistic regression OR, the risk of a systolic pressure greater than 140 mmHg was 2.30 (95% CI 1.80–4.46) for the study girls, but was not significantly increased for the study boys [119].

Additional trials followed; among others, an investigation in 4096 Norwegian girls 13–19 years old, confirmed that maternal PE was associated with higher SBP (2.9 mmHg difference, *p* < 0.001), as well as DBP (1.7 mmHg difference, *p* = 0.001) [120]; and an Australian study of 2608 mother-offspring pairs followed for 21 years [121], showing that, at age 21, offspring of women who had HDP, had 3.46 mmHg greater SBP and 3.02 mmHg greater DBP.

A first metanalysis of the effects of PE on BP in offspring was carried out in 2009 by Ferreira et al. [122] who selected seven articles, all published between 1980 and 2009. They found that offspring of women with PE had 2.3 mmHg (95% CI: 1.6–3.0) higher SBP levels compared with those from normal pregnancies; there was also an elevation in DBP of 1.7 mmHg (95% CI: 0.9–2.4).

The pooled mean difference in SBP was not affected by heterogeneity (I^2^¼ 0%, P¼ 0.852), but this may have affected the mean difference in DBP, where about 43% of the total variance across studies was due to heterogeneity rather than chance alone (P¼ 0.071).

A new, systematic review and meta-analysis on the subject is being carried out. So far, only the protocol of this study has been published; from which it can be inferred that it will provide high-quality evidence of the effects in offspring BP of different subtypes of HDP [123].

### 4.2. Cardiovascular Diseases

In 1995, Barker [124] proposed that fetal under nutrition in middle to late gestation leads “to disproportionate fetal growth”, programming for “later coronary heart disease”.

This field has now been explored by a variety of investigations that have further clarified the situation. In 2003, Tenhola et al. [125] published the results of a comparative investigation in two groups, each of sixty 12-year-old children, born after a normal or a preeclamptic pregnancy. They found that PE children had significantly higher mean systolic (116.4 vs. 113.2 mmHg; *p* = 0.021) and diastolic (73.9 vs. 70.3 mmHg; *p* = 0.022), even after adjusting for weight and height. At 12 years of age, systolic BP values correlated inversely with birth weight (r = −0.459; *p* < 0.001) and length SD scores (r = −0.429; *p* = 0.001) in the PE children. At the time, the authors concluded that it was not known whether this phenomenon was due to genetic factors or to PE.

Over the last decade, a number of studies have attempted to clarify the situation: Timpka et al. [126], extracting data from the Avon Longitudinal Study of Parents and Children prospective population-based birth cohort study [127], found 1592 offspring who underwent echocardiography at a mean age of 17.7 ± 0.3 and evaluated possible differences in those born from mothers with HDP. They observed that exposure to maternal PE and to gestational hypertension was associated with greater relative wall thickness (OR: 0.025; 95%CI, 0.008–0.043 and 0.010; 95%CI, 0.002–0.017). In addition, PE, but not pregnancy hypertension, was also associated with a smaller left ventricular end-diastolic volume (−9.0 mL; 95%CI, −15/−3.1). They concluded that adolescents who had been exposed in utero to PE had early signs of concentric heart remodeling, possibly affecting future cardiac function, as well as the risk of CVD later in life.

Palmsten et al. [128], in a birth cohort from New England, observed that HDP was associated with hypertension in offspring later in life (from 8.8% to 17.4%; aOR: 1.88, 95%CI: 1.00–3.55). In addition, this association increased to an aOR: 1.97 (95%CI: 1.04–3.72), after excluding offspring of women who reported hypertension during pregnancy only.

Finally, a just-published, large, retrospective, controlled cohort study in the USA assessed the risk for early mortality among offspring of pregnancies complicated by HDP and PE over a period of 20 years (1947 to 1967) [129]. They found that mortality risks for metabolic, respiratory, digestive, nervous and external causes of death did not differ between exposed and unexposed groups. In contrast to this, mortality rates from CVD were greater in exposed than in unexposed offspring (aHR: 1.57; 95%CI 1.16–2.12). There were two important observations in this study: the first dealt with sex differences in mortality risk: this was increased in exposed male (aHR: 1.92; 95% CI: 1.27–2.88), not in female subjects (aHR: 0.97; 95% CI: 0.81–1.94). The second had to do with the additive nature of the effect: there was a significant effect (P¼ 0.047) of birth order on CVD mortality.

In conclusion, the available evidence shows a predisposition to CVD, and a higher incidence of cardiovascular risk factors among children born to preeclamptic mothers. In addition, both experimental models and human epidemiological studies have shown that infants of pregnancies complicated by PE have an increased risk of developing high BP, CVD (independent of PTB) and stroke in later life [130].

**Figure 2:**
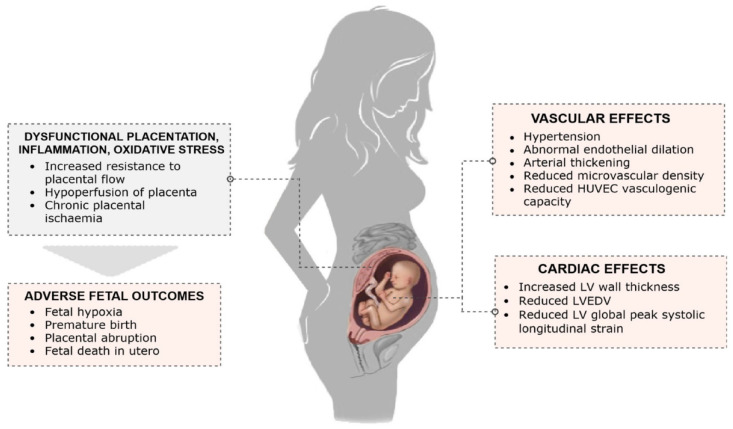
Effects of Preeclampsia on the fetus. HUVEC stands for human umbilical vein. ED; LV, left ventricle; LVEDV, left ventricular end-diastolic volume. Reproduced from: Fox et al., 2019.

**Figure 3:**
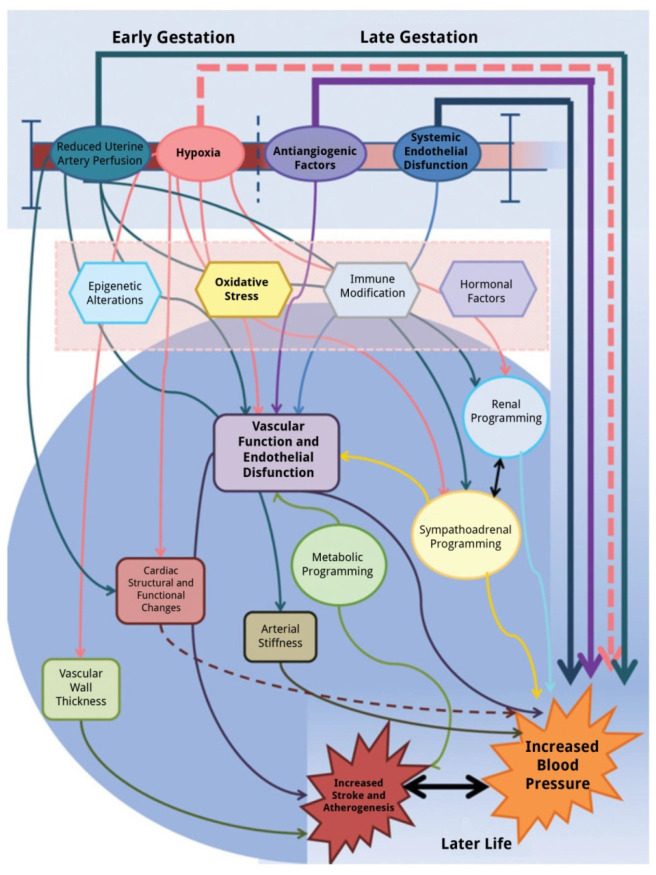
Summary of the potential and demonstrated mechanisms by which in utero exposure to pre-eclampsia may lead to altered cardiovascular physiology and risk in the offspring in later life. Reproduced from: Davies et al., 2012.

A graphic representation of the effects of PE on the fetus is presented in Figure 2 [131], and the potential mechanisms of cardiovascular programming in a fetus following a preeclamptic pregnancy are summarized in Figure 3 [79].

### 4.3. Possible Pathogenic Mechanisms of the Effects on the Newborn

Based on the ‘Barker hypothesis’, it is likely that intrauterine growth restriction can in itself play an important role in determining cardiovascular risk and specifically, myocardial disease. However, other factors will also influence the development later in life of hypertension, coronary and myocardial disease [132]. These factors include: ED; genetic disposition and epigenetic modifications; these are detailed below. In addition, concurrent intrauterine exposures, and the lifestyle of the affected individual, may also play a role.

#### 4.3.1. Endothelial Dysfunction

In 2010, Lazdam et al. [133] published the results of a 20-year follow-up study of 71 preterm subjects, of whom 19 were born to a hypertensive and 52 to a normotensive pregnancy. These two groups were then compared with 38 subjects born at term following uncomplicated pregnancies. They observed that offspring born preterm to either hypertensive or normotensive pregnancy had higher peripheral and central blood pressure compared to subjects born full-term. Central mean arterial pressure after preterm hypertensive pregnancy was 84.927.0 mmHg; after preterm normotensive pregnancy, 84.138.9 mmHg; and after full-term pregnancy, 76.247.96 mmHg; *p* = 0.0009). Importantly, underlying vascular phenotypes were different, with preterm offspring of normotensive pregnancy showing a greater arterial stiffness than offspring of hypertensive pregnancy (5.920.84 vs. 5.420.73 m/s; *p* = 0.039). In addition, offspring of hypertensive pregnancies had greater carotid intima-media thickness (0.520.04 vs. 0.480.06 mm; *p* = 0.013) and a 30% lower flow-mediated dilatation (4.254.02% vs. 6.794.38%; *p* = 0.05). They concluded that, on the one hand, prematurity is associated with elevated blood pressure in later life; on the other, the predominant underlying vascular phenotype depends on maternal pathology.

Kvehaugen et al. [68] found that endothelial function is significantly reduced in both mothers and children following a pregnancy complicated by PE combined with Small for Gestational Age infants (mothers: *p* < 0.001; children: *p* < 0.05). Importantly (see Section 4.3.2.), post-partum maternal soluble fms-like tyrosine kinase 1 (*p* = 0.05) and hs-CRP (*p* = 0.02) were elevated in the PE group, persisting at 5 to 8 years (*p* < 0.05), accompanied by increased inflammatory and antiangiogenic maternal biomarkers. This is a clear indication that there is a trans-generational risk of CVD after PE. Similar results were found by Giachini et al. [134] in Latin American women.

Starting from the hypothesis that during PE, vasculotoxic factors are released from the placenta into the maternal circulation, Jayet et al. [135] compared Pulmonary Artery Pressure (PAP) and Flow-Mediated Dilation (FMD) of the brachial artery in 48 offspring of women with PE and 90 offspring of women with normal pregnancies. In order to enhance the chances of detecting the phenomenon, all their subjects were born and lived permanently at an altitude of 3600 m. They observed a PAP roughly 30% higher (mean SD: 32.15.6 vs. 25.34.7 mmHg; *p* = 0.001) and an FMD, some 30% smaller (6.31.2% vs. 8.31.4%; *p* = 0.0001) in offspring of mothers with PE than in controls, with a strong inverse relationship between FMD and PAP. Results led to the conclusion that PE leaves a persistent defect in both the systemic and pulmonary circulation of the offspring, predisposing to exaggerated hypoxic pulmonary hypertension already during childhood.

Some insight into the mechanism through which PE predisposes to early hypertension in offspring has been provided by Yu et al. [136], who explored the potential epigenetic regulation of delta-like homolog 1-maternally expressed gene 3 region, in human umbilical vein ED, and its connection with endothelium-derived factors.

They recruited 67 singletons from preeclamptic gestations, compared them to 58 singletons born from normal pregnancies and found that *DBP* was significantly lower in preeclamptic offspring born over 34 weeks, compared with normal offspring (53.59 ± 1.38 vs. 59.9 ± 1.40 mmHg, *p* < 0.01), with a consequential higher pulse pressure difference. Quantitative real-time PCR documented that the level of imprinted gene *DLK1* increased significantly and *MEG3* level decreased in umbilical vein ED of the PE group. This was associated to a lower expression of endothelial nitric oxide synthase and VEGF, higher expression of endothelin-1. They concluded that an altered expression of imprinted genes *DLK1* and *MEG3* was caused by hypermethylation of *IG-DMR* in umbilical vein ED of PE group, accompanied by lower secretion of nitrite, VEGF, and higher secretion of endothelin-1.

#### 4.3.2. Genetic Alterations

In 2015, Hromadnikova et al. [137] observed that the expression profile of microRNAs differed between subjects with a normal pregnancy and those in whom complications occurred and suggested that epigenetic changes induced by pregnancy-related complications in placental tissue may cause later onset of cardiovascular and cerebrovascular diseases in offspring. They hypothesized that a sudden onset of hypertension during pregnancy may result in misguided biological programming of the fetus, via changes in the epigenome, resulting in suboptimal infant development.

In this regard, there is evidence that in pregnancies complicated by PE, impaired placental perfusion coupled to oxidative stress, ED and immune modifications can disturb epigenetic programming in offspring, resulting in derangements of their vascular epigenome and function [94,138]. Herzog et al. [75] have examined tissue-specific genome-wide DNA methylation in placentas and umbilical cord endothelial and white blood cells of women with PE. They observed a series of differences in the methylation process in these specimens, situated in or close to genes associated with cardiovascular-metabolic developmental pathways. They found also evidence that oxidative stress may produce more consequences in pregnancies with early-onset PE, in which placental and newborn tissues are subjected to it from early pregnancy onwards. They hypothesized that in this situation, the altered epigenetic programming, rather than being a consequence of the disease, is in fact already ongoing, possibly inducing the further development of PE.

To evaluate a possible association between either HDP (10 cohorts) or PE (3 cohorts) and epigenome-wide DNA methylation in cord blood, Kazmi et al. [139] performed meta-analyses within the Pregnancy and Childhood Epigenetics Consortium. They found that epigenome-wide associations of HDP with offspring DNA methylation were modestly consistent with the equivalent epigenome-wide associations of PE. These results suggest that genes located at/near HDP-associated sites may be involved in developmental, embryogenetic, or neurological pathways.

This information will help in designing studies aimed at elucidating the associations between prenatal PE exposure and the CVD risk in offspring.

There is also some experimental evidence on the mechanisms through which these effects can be induced: Herzog et al. [140] determined that PE is associated with a smaller umbilical cord vein area and wall thickness, independent of gestational age and birth weight. They suggested that this phenomenon “may serve as a proxy of disturbed cardiovascular development in the newborn”.

As mentioned above, novel key data were obtained from the Scandinavian Genome-wide association study of offspring from pregnancies complicated by PE [96,97].

Whereas this anomaly provides a genetic basis to explain the risk of PE, it is not known whether it may influence future risks for the newborn.

#### 4.3.3. Comparison of Biochemical and Biophysical Markers in Offspring

Of great importance for improving the management of offspring of pregnancies complicated by PE is to carry out comparative studies (offspring of PE women, adolescent, adult, women with HDP). Unfortunately, our search of the literature could not identify any such investigation.

## 5. Conclusions

In a just published editorial, Henry & Canoy [141] stressed that it is today well-established that mothers with HDP have increased cardiometabolic health risks long after delivery. The existence of these adverse effects is proven by both retrospective and prospective cohort studies, consistently finding after HDP, 1.5–3 times increase in risk of a wide range of cardiovascular conditions.

The underlying mechanisms remain uncertain; however, evidence points to impaired endothelial function during and after preeclamptic pregnancies, as well as structural abnormalities, including increased carotid intima-media thickness and accelerated coronary calcification and plaque deposition.

Key to the identification of these mechanisms is a proper understanding of the relationship between the immune system and a variety of diseases. In this respect, a number of hypotheses have been proposed, but no real conclusions have been reached. An important point has emerged so far: immune reactions have desirable and undesirable consequences on their final outcome, therefore playing a novel critical role in the pathogenesis [142].

For a long time, pregnancy has been considered possible thanks to the existence of a “closed container”: the maternal uterus was considered a place where immune cells had no access, enabling the acceptance of paternal alloantigens. Today, this concept has been completely revolutionized: a successful gestation and delivery are the result of a fine and strongly regulated balance among maternal immune cells [143]. In this respect, the described phenomenon of defective deep placentation may alter this balance, leading to a number of pathological conditions [9]. The process leads to acute atherosis and atherosclerosis and, in view of the fact that atherogenesis is the primary driver of CVD, the hypothesis can be formulated that these lesions represent the main cause of long-term consequences of cardiovascular pathology in the mother and the offspring.

As pointed out by Lazdam et al. [144], it is now becoming clear that PE is more than an isolated disease of pregnancy. The long-term health implications of this condition for both the women and their children are increasingly being recognized and incorporated into clinical risk assessments. Both women and children exposed to PE exhibit an adverse vascular phenotype, a propensity to subclinical atherosclerosis, and increased risk of adverse cardiac and vascular events in future life.

Of importance for the understanding of the long-term consequences is the recognition that the main cause of the atherosclerotic process is inflammation: though multifactorial and with multiple risk factors, the primum movens of atherosclerosis is an inflammatory reaction that can be triggered by a variety of antigens [77].

**Figure 4:**
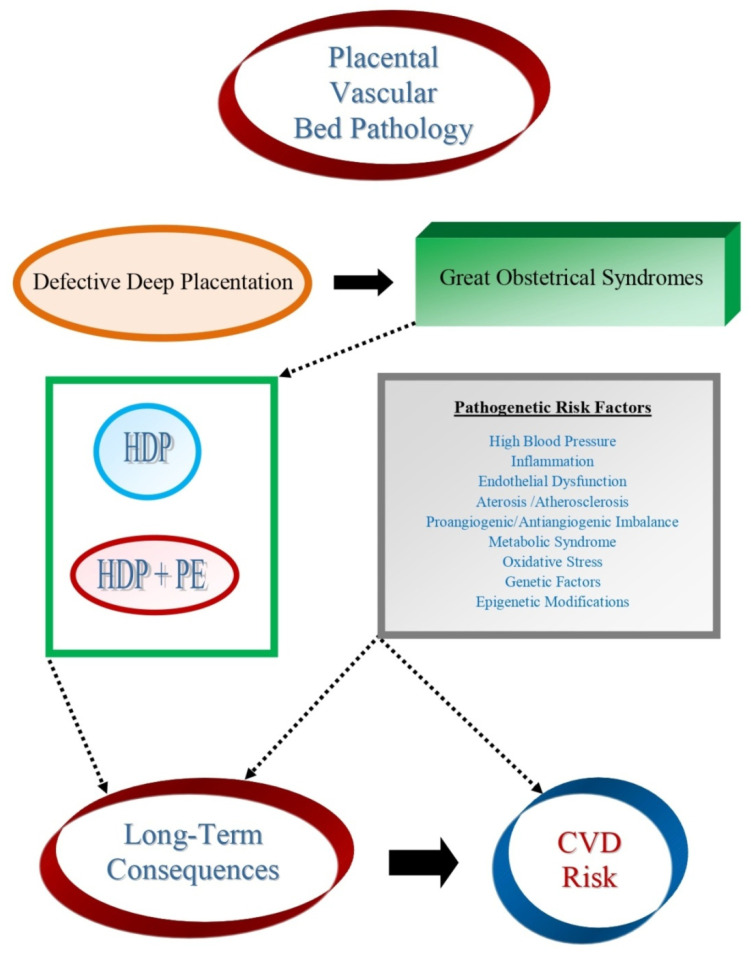
Placental vascular bed pathology is at the core of defective deep placentation which leads virtually to all Great Obstetrical Syndromes. In particular, hypertensive disease in pregnancy (HDP) and its association with preeclampsia (PE) share a series of risk factors with cardiovascular diseases (CVD) that may be responsible for long term consequences of placental vascular bed pathology and the increased incidence of CVD risk among mothers and their off-springs.

A successful pregnancy is the result of a fine balance between the maternal immune system and the presence of paternal alloantigens in the fetus [143]. When the balance is upset, inflammatory reactions can take place, triggering a number of processes leading to placental bed vascular pathology, the common denominator of the “Great Obstetrical Syndromes” [8,36]. In this contest, inflammatory mediators play a crucial role in modifying the general homeostasis of the vascular bed, triggering profound changes and alterations in the role of cells’ functions. Additional risk factors, such as hypertension, not only contribute to the gestational vascular bed pathology, but represent important risk factors for future CVD in pregnant women and in their offspring (Figure 4).

Epigenetic programming is essential for lineage differentiation, embryogenesis and placentation in early gestation. Epigenetic association studies of tissue-specific genome wide DNA methylation have now been conducted in various tissues including the placenta. This is a unique structure because, given its very short life span, it undergoes rapid growth and dynamic, structural and functional changes [145]; this offers a unique opportunity to investigate the atherosclerotic process.

Hopefully, new, advanced investigations will provide clear evidence on how modifications in placental processes can affect future CVD risk. Furthermore, since the placenta, although developing within the mother, possesses a genome identical to that of the fetus, this organ may represent an important model for the study of epigenetic changes in the offspring and identify potential CVD risk factors. A point worth mentioning is that most published information on health after HDP is from high-income country populations, whereas burden of the short and long-term consequences falls disproportionately on low and middle-income countries. This calls for clear guidelines and Gamble et al. [146] have recently reviewed existing guidelines, finding that they recommend that stakeholders be made aware of the risk of long-term consequences. To prevent them there should be a yearly provision of monitoring of BP, and a periodical check of renal functions, urinalysis and lipid profile. This, alongside with lifestyle modifications and monitoring of possible development of CVD.

It can be concluded that there is a need to define the trajectory of women with a history of placental vascular pathology and HDP. This, however, requires the identification of candidate biomarkers that should represent the best approach to the development of effective screening, risk stratification, and preventive measures.

## Figures and Tables

**Table 1:** Association of adverse pregnancy outcomes with risk of diabetes or risk factors for coronary heart disease and vascular disease. Great Obstetrical syndromes are strictly connected to adverse long-term health consequences. Women with a history of adverse pregnancy outcome appear to be at increased risk of metabolic and vascular diseases in later life.

Pregnancy Outcome	Incidence in Pregnancy %	Risk Factors Shown to be Perturbed after Pregnancy	Association or Risk Ratio (95% Cl)
Gestational diabetes	1.9–5.0	Lipids	Increased risk for type 2 diabetes, especially if recurrence of gestational diabetes in a subsequent Pregnancy. No data on Coronary heart disease
		Blood pressure	
		Large vessel function	
		Small vessel function	
Preeclampsia (PE)	2–4	Lipids	1.9 (1.0–3.5) vs. pregnancy
		Clotting	induced hypertension alone
		Fasting insulin	1.7 (1.3–2.2) vs. no-PE
		Large vessels function	2.0 (1.5–2.5) vs. no-PE
Low birth weight (<2500 g)	5	Not studied	11.3 (2.5–36.1) vs. ≥3500 g
			7.1 (2.6–18.7) vs. ≥3500 g
Preterm delivery (<37 weeks)	5–6	Not studied	1.8 (1.3–2.5) vs. term deliv
			2.1.(1.2–3.5) vs. term deliv

Modified and reproduced with permission from: Sattar and Greer 2002.

**Table 2:** Biochemical cardiovascular risk factors after hypertensive pregnancy disorder.

The Following Modifications in Values for the Parameters Investigated Were Observed:	
Glucose:	+0.17 mmol/L (95% CI: 0.08–0.25 mmol/L)
Insulin:	+3.46 mU/mL (95% CI: 2.34–4.58 mU/mL)
Triglycerides:	+0.13 mmol/L (95% CI: 0.05–0.21)
Total cholesterol:	+0.22 mmol/L (95% CI: 0.11–0.33 mmol/L)
HDL-cholesterol:	−0.11 mmol/L (95% CI: −0.18 to −0.04 mmol/L)
LDH-cholesterol:	+0.21 mmol/L (95% CI: 0.10–0.32)

All these changes indicate that hypertensive pregnancy disorders place a woman at an increased risk of cardiovascular diseases later in life. Data are from: Hermes et al., 2012.

## Data Availability

Not applicable.
